# Multiplexity versus correlation: the role of local constraints in real multiplexes

**DOI:** 10.1038/srep09120

**Published:** 2015-03-13

**Authors:** V. Gemmetto, D. Garlaschelli

**Affiliations:** 1 Instituut-Lorentz for Theoretical Physics, Leiden Institute of Physics, University of Leiden, Niels Bohrweg 2, 2333 CA Leiden, The Netherlands

## Abstract

Several systems can be represented as multiplex networks, i.e. in terms of a superposition of various graphs, each related to a different mode of connection between nodes. Hence, the definition of proper mathematical quantities aiming at capturing the added level of complexity of those systems is required. Various steps in this direction have been made. In the simplest case, dependencies between layers are measured via correlation-based metrics, a procedure that we show to be equivalent to the use of completely homogeneous benchmarks specifying only global constraints. However, this approach does not take into account the heterogeneity in the degree and strength distributions, which is instead a fundamental feature of real-world multiplexes. In this work, we compare the observed dependencies between layers with the expected values obtained from maximum-entropy reference models that appropriately control for the observed heterogeneity in the degree and strength distributions. This information-theoretic approach results in the introduction of novel and improved multiplexity measures that we test on different datasets, i.e. the International Trade Network and the European Airport Network. Our findings confirm that the use of homogeneous benchmarks can lead to misleading results, and highlight the important role played by the distribution of hubs across layers.

The study of networks allows scientists to suitably represent and analyze biological, economic and social systems as a set of units (nodes) connected by edges (links) symbolizing interactions^1^,^2^,^3^,^4^.

However, this approach may actually lead to an oversimplification: indeed, several systems are composed by units connected by multiple kinds of interaction. In such systems, the same set of nodes is joined by various types of links, each of those representing a different mode of connection^5^. The simplest way to analyse such systems is the aggregation of the various levels in a single network, but it turns out that such a simplification may discard fundamental information about the real topology of the network and therefore about possible dynamical processes acting on the system^6^. For instance, such an aggregation may result in a loss of information about the distribution of the hubs across layers, which is instead crucial for the control of several processes arising on an interdependent network^7^. Then, in order to solve such an issue, in the last few years the study of multi-layer networks has been pursued. In this context, new quantities aiming at mathematically analyzing multi-level networks have been provided^8^,^9^,^10^,^11^; furthermore, models of growth^12^,^13^,^14^ and dynamical processes occurring on multiplexes, such as epidemic spreading^15^, diffusion^16^, cooperation^17^ and information spreading^18^ have been designed.

In this work, we follow the path towards the definition of measures that can be applied to multi-level networks, in order to characterize significant structural properties of these systems, in particular focusing on the analysis of the dependencies between layers. We argue that, in order to properly characterize such dependencies, a comparison between the observed correlation and some notion of expected correlation is required. We therefore exploit the concept of multiplex ensemble^19^,^20^,^21^, aiming at the definition of suitable null models for multi-layer complex networks, in order to compare the observed overlap between layers with the expected overlap one would find in a random superposition of layers with the same node-specific properties. In particular, since our purpose is precisely that of measuring such dependencies, we will consider uncorrelated multiplex ensembles, in order to define a null model for the real system so that it is possible to compare the observed correlations with reference models where the overlap between layers is actually randomized and, at the same time, important node-specific properties of the real network are preserved.

Various efforts have already been made about the study of correlations in multi-level networks^22^,^23^,^24^, but the comparison of the observed results with the expected ones has generally been based on a - sometimes implicit - assumption: the benchmark was a completely homogeneous graph. In particular, here we show that correlation-based measures of inter-layer dependency (of the type used e.g. in Ref. 22) build on an implicit assumption of homogeneity, which in the unweighted case is equivalent to the choice of the Random Graph as null model. Similarly, for weighted networks, the chosen benchmark was equivalent to the Weighted Random Graph, where the weight distribution is independent from the considered pair of nodes^25^.

However, this assumption of uniformity in the probability distributions strongly contrasts with the observed findings in real-world complex systems. Indeed, one of the most well-known features of complex networks is their heterogeneity^26^, both in the degree distribution and in the strength distribution; it is therefore crucial to take this aspect into account when proper null models for graphs are designed. Moreover, it has been recently shown that, in multiplex networks, the correlation between degrees (and strengths) of nodes across different layers is also an important structural feature that can have strong effects on the dynamics^7^,^27^. Ultimately, such inter-layer degree correlations determine the distribution of hubs across layers, i.e. whether the same nodes tend to be hubs across many layers, or whether different layers are characterized by different hubs. We therefore aim at measuring multiplexity in terms of the “residual” inter-layer dependencies that persist after we filter out, for each layer separately, the effects induced by the heterogeneity of the empirical degree (for unweighted networks) or strength (for weighted graphs) distribution. We show that such a refinement can completely change the final findings and lead to a deeper understanding of the actual dependencies observed between layers of a real-world multiplex.

First, we introduce a new “absolute” measure of multiplexity designed to quantify the overlap between layers of a multi-level complex network. Second, we derive the expression of the expected value of such a quantity, both in the binary and in the weighted case, for randomized networks, by enforcing different constraints. Third, we combine the “absolute” multiplexity and its expected value into a filtered, “relative” measure of multiplexity that has the desired properties. We finally apply our measures to two different real-world multiplexes, namely the International Trade Network and the European Airport Network, showing that the analysis of the dependencies between layers can actually make some important structural features of these systems explicit.

Indeed, while the former shows significant correlations between layers (i.e., traded commodities), in the latter almost no overlap can generally be detected, thus clearly defining two opposite classes of multiplexes based on the observed correlations. Furthermore, we will link such a behaviour with the distribution of the hubs across layers, hence providing a straightforward explanation to the observed findings.

## Results

### Null models

It is possible to design null models for multi-level networks as maximum-entropy ensembles on which we enforce a given set of constraints^21^. In particular, we exploit the concept of uncorrelated multiplex ensemble, so that the definition of proper null models for the considered multiplex reduces to the definition of an indipendent null model for any layer of the system. In order to do this, we take advantage of the concept of canonical network ensemble, or exponential random graph^28^, i.e. the maximum-entropy family of graphs satisfying a set of constraints on average. In this context the resulting randomized graph preserves only part of the topology of the considered real-world network and is entirely random otherwise, thus it can be employed as a proper reference model. However, fitting such previously defined models to real datasets is hard, since it is usually computationally demanding as it requires the generation of many randomized networks whose properties of interest have to be measured.

In this perspective, we exploit a fast and completely analytical Maximum Entropy method, based on the maximization of the likelihood function^29^,^30^,^31^, which provides the exact probabilities of occurrence of random graphs with the same average constraints as the real network. From such probabilities it is then possible to compute the expectation values of the properties we are interested in, such as the average link probability or the average weight associated to the link established between any two nodes. While the adoption of such a method is not strictly required when dealing with global constraints like the total number of links observed in a network (the so-called Random Graph), it becomes crucial when facing the problem of enforcing local constraints such as the degree sequence or the strength sequence (Binary or Weighted Configuration Model). More information about such null models can be found in the Methods Section and in the Supplementary Information.

Before introducing our measures of multiplexity, we make an important preliminary observation. Simple measures of inter-layer dependency are based on correlation metrics, which in turn rely on an assumption of uniformity, such assumption being ultimately equivalent to the choice of a uniform Random Graph as a null model. We illustrate this result in more detail in the Supplementary Information.

### Multiplexity

When unweighted networks are considered, we define the “absolute” binary multiplexity between any two layers *α* and *β* as:
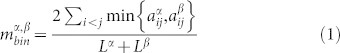
where *L^α^* is the total number of links observed in layer *α* and 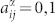 depending on the presence of the link between nodes *i* and *j* in layer *α*. Such a quantity represents a normalized overlap between any pair of layers and can therefore be thought of as a normalized version of the global overlap introduced in Ref. 21.

The previous definition can be easily extended to weighted multiplex networks. We define the “absolute” weighted multiplexity as:
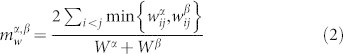
where 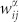 represents the weight of the link between nodes *i* and *j* in layer *α* and *W^α^* is the total weight related to the links in that layer. Both (1) and (2) range in [0, 1], are maximal when layers *α* and *β* are identical - that is, if there is complete similarity between those two layers - and minimal when they are fully uncorrelated; in this perspective, they evaluate the tendency of nodes to share links in different layers.

However, the above absolute quantities are uninformative without a comparison with the value of multiplexity obtained when considering a null model. We may indeed measure high values of multiplexity between two layers due to the possibly large observed values of density, without any significant distinction between real dependence and overlap imposed by the presence of many links in each layer (thus forcing an increase in the overlap itself).

Furthermore, we cannot draw a clear conclusion about the amount of correlation between layers by just looking at the observed value, since such a measure is not universal and, for instance, no comparison between different multiplexes can be done based on the raw “absolute” multiplexity.

We therefore introduce the following “relative” or rescaled quantity along the lines of Refs. 34, 35:
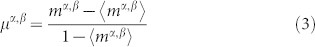
where *m^α^*^,*β*^ is the value measured for the observed real-world multiplex and 〈*m^α^*^,*β*^〉 is the value expected under a suitably chosen null model. The main null models that we will consider are respectively the Random Graph (RG) and the Binary Configuration Model (BCM) in the unweighted case, the Weighted Random Graph (WRG) and the Weighted Configuration Model (WCM) in the weighted case. We will characterize them in more detail in the Methods Section and in the Supplementary Information.

This rescaled quantity is now directly informative about the real correlation between layers: in this context, since *μ^α^*^,*β*^ ∈ [−1, 1], positive values represent positive correlations, while negative values are associated to anticorrelated pairs of layers; furthermore, pairs of uncorrelated layers show multiplexity values comparable with 0.

One of the motivations of the present work is the consideration that, in the binary case, when the Random Graph is considered as a null model, the previous quantity (3) can actually be reduced to the standard correlation coefficient between the entries of the adjacency matrix referred to any two layers *α* and *β* of a multi-level graph, defined as:
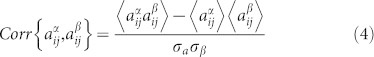


In the Supplementary Information, we show that the previous expression is nothing but a different normalization of the rescaled binary multiplexity defined in (3):
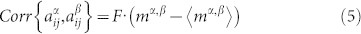
where *F* is a factor depending on *L^α^*, *L^β^* and *N*.

### Binary analysis

We validate our definitions applying them to two different real-world multiplexes: the International Trade Network (*N* = 207 countries, *M* = 96 layers representing traded commodities), available as a weighted multi-level network, and the European Airport Network (*N* = 669 airports, *M* = 130 airlines), provided as an unweighted system. A detailed description of the datasets can be found in the Supplementary Information.

**Figure 1:**
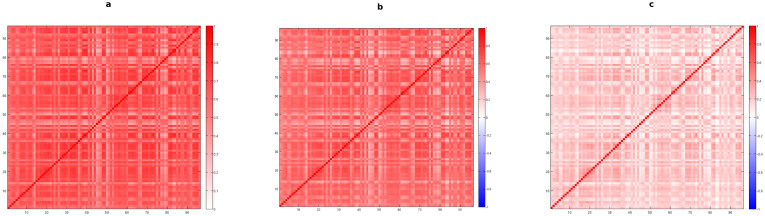
Analysis of the binary multiplexity between layers of the International Trade Network in 2011. Colorcoded matrices with entries given by *m_bin_* (a), *μ_RG_* (b) and *μ_BCM_* (c) for any pair of layers (commodities).

The implementation of the concept of multiplexity to different networks can lead to completely divergent results, according to the structural features of the considered systems. Indeed, the application of (1) to the International Trade Network leads to the color-coded multiplexity matrix shown in Figure 1(a). Such an array generally shows very high overlaps between layers, i.e. between different classes of commodities, pointing out that usually each country tends to import from or export to the same set of countries almost independently from the traded items; this is true in particular for most of the edible products (layers characterized by commodity codes ranging from 1 to 22, as listed in the Supplementary Information).

In order to have a complete picture of the dependencies between layers of the considered systems, we have to compare our findings with the overlaps expected for multiplexes having only some of the properties in common with the observed ones. The simplest benchmark, as well as the most widely used, is the Random Graph, which discards, as we said, any kind of heterogeneity in the degree distributions of the layers. When we compute *μ_RG_* for the International Trade Network, we obtain the multiplexity matrix shown in Figure 1(b). The plot clearly shows that most of the correlations are still present: this layer-homogeneous null model, together with the presence of comparable densities across the various layers, does not significantly affect the expected overlaps. So far, we have discarded heterogeneity in our null models. However, this can considerably affect the significance of our findings. Therefore, we introduce heterogeneity in the degree distribution within the reference model by means of the previously defined Configuration Model. This way, it is actually possible to detect only the non-trivial dependencies, therefore discarding all the overlaps simply due to the possibly high density of the layers, that would otherwise increase the observed interrelations even if no real correlation is actually present.

This is exactly what happens when the World Trade Network is analyzed. Indeed, as shown in Figure 1(c), we find out that a significant amount of the binary overlap observed in this network is actually due to the information included in the degree sequence of the various layers, rather than to a real dependence between layers. This method is therefore able to detect the really meaningful similarity between layers, discarding the trivial overlap caused by the presence, for instance, of nodes having a high number of connections in most of the layers. This non-significant overlap is thus filtered out by our procedure. Such observations clearly show that the Random Graph is not the most proper reference model in order to obtain an appropriate representation of crucial properties of such multi-level systems.

We now note that linear correlations have been used in the literature to produce dendrograms^22^,^36^. As we mentioned, the use of linear correlations corresponds to the choice of the Random Graph as null model. Here, we can instead make use of *μ_BCM_* to implement an improved hierarchical clustering procedure, as reported in the Supplementary Information.

**Figure 2:**
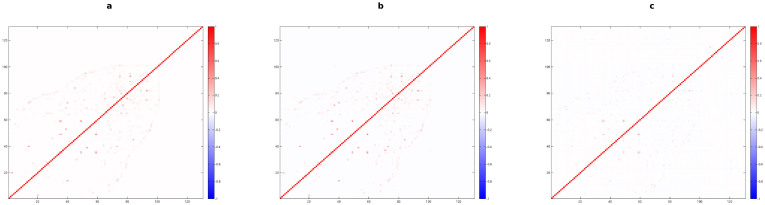
Analysis of the binary multiplexity between layers of the European Airport Network. Color-coded matrices with entries given by *m_bin_* (a), *μ_RG_* (b) and *μ_BCM_* (c) for any pair of layers (airlines).

A completely different behaviour can be observed for the European Airport System. Indeed, low values of multiplexity observed for such a network (Figure 2(a)) illustrate nearly no overlap between most of the layers: this highlights the well-known tendency of airline companies to avoid superpositions between routes with other airlines.

In Figure 2(b) we show the residual correlations obtained after the application of the Random Graph: almost no difference can be perceived with respect to Figure 2(a), since the expected overlap in this case is very small, due to the very low densities of the various layers. We should point out that the Random Graph is not a proper reference model for this real-world network, since the assumption of uniformity in the degree of the different nodes (i.e., airports) is actually far from the observed structure of such a system, as we will highlight later. Nevertheless, in Figure 2(c) we show that, at first glance, the adoption of the Configuration Model does not look strictly required when the European Airport Network is considered, except for a more suitable mathematical approach, since the overall matrix looks apparently similar to the previous Figure 2(b). However, the presence of a larger number of negative values of multiplexity and the simultaneous disappearance of most of the significantly high values highlight once more the anti-correlated character of such a system, and this crucial structural property of the airport multiplex network was not fully revealed by the application of the Random Graph.

In this case, a dendrogram designed form matrices reported in Figure 2 would not be meaningful, since most of the layers meet at a single root level, due to the very low correlation observed between them.

### Weighted analysis

Since the International Trade Network is represented by a weighted multiplex, the analysis of weighted overlaps between layers of that system can be performed, in order to obtain more refined information about the dependencies between different classes of commodities. We should indeed point out that, for the World Trade Web, while the binary overlaps provided by (1) only supply information about the dependencies between the topologies of the various layers representing trade in different commodities, the weighted multiplexity defined in (2) is able to detect patterns of correlation between quantities of imported and exported classes of items. In this perspective, observing high correlations is therefore more unlikely. This is due, mathematically, to the functional form of the definition of the multiplexity given in (2), which is significantly dependent on the balance between weights of the corresponding links in different layers; such a property, therefore, tends to assign higher correlations to pairs of commodities characterized by similar global amount of trade, as we want.

**Figure 3:**
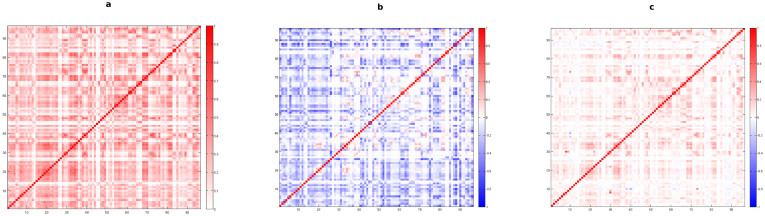
Analysis of the weighted multiplexity between layers of the International Trade Network in 2011. Color-coded matrices with entries given by *m_w_* (a), *μ_WRG_* (b) and *μ_WCM_* (c) for any pair of layers (commodities).

In Figure 3(a) we show the color-coded matrix associated to the raw values of weighted multiplexity as observed in the International Trade Network: clear dependencies between different layers are still present, but a comparison with its corresponding binary matrix (shown in Figure 1(a)) explicitly reveals that, while some pairs of layers are significantly overlapping, several pairs of commodities are now actually uncorrelated, as expected when the weights of the links are taken into account.

In order to provide information about the relation between the observed dependencies between layers and the expected ones under a given benchmark, as a first estimate, we calculate *μ_WRG_*, therefore considering the corresponding Weighted Random Graph as a reference for our real-world network. Our findings show, in Figure 3(b), a strongly uncorrelated behavior associated to most of the pairs of commodities, in contrast with our intuitive expectations based on the results obtained in the binary case.

We then compare the observed multiplexity with its expected values under the Weighted Configuration Model. Results, shown in Figure 3(c), exhibit a completely different behavior with respect to Figure 3(b), thus highlighting once more the importance of taking into account the heterogeneity in the weight and degree distributions within the considered null model. Indeed, we observe that, exploiting this more suitable reference, several pairs are still correlated, even in the weighted case, some of them are actually uncorrelated, as expected by looking at the corresponding binary matrix (Figure 1(c)), and only a few, with respect to the Weighted Random Graph case, remain anti-correlated. In general, however, the dependencies between layers in the weighted case are less noticeable, as we can see from a comparison between the matrices shown in Figures 1(c) and 3(c).

### Hubs distribution

**Figure 4:**
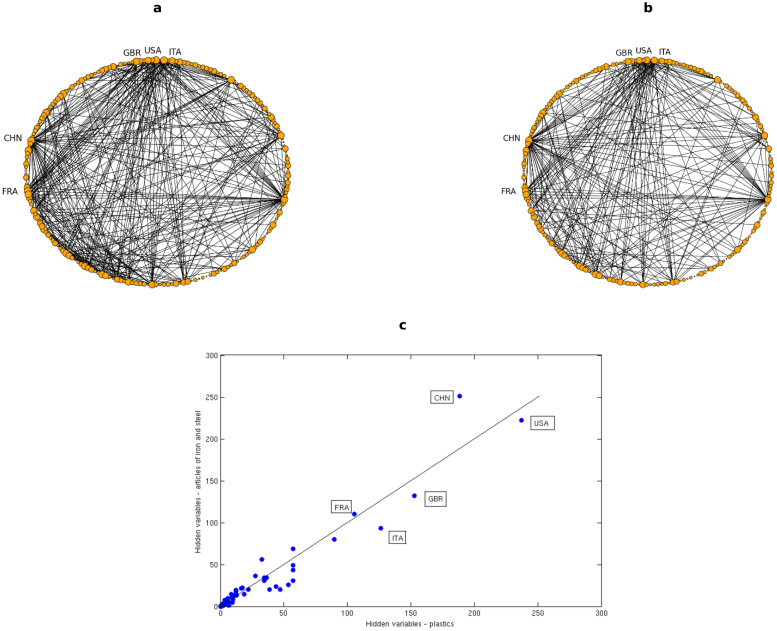
Hubs distribution in the International Trade multiplex. Top panels: graphs representing two layers of the system, respectively those associated to trade in plastic (a) and articles of iron and steel (b); nodes represent trading countries; size of a node is proportional to its degree in that layer. Only links associated to a trade larger than 100 millions dollars are reported. Bottom panel: scatter plot of the hidden variables *x_i_* relative to each of the nodes for the same two layers; the black line represents the identity line.

The different behaviours observed for the two considered multiplexes can be, at least partly, explained in terms of distribution of the hubs across layers. As we show in Figure 4(a) and 4(b), generally any two layers of the World Trade Network exhibit the same set of hubs (which in this particular case are represented by the richest and most industrialized countries). Indeed, the two network layers plotted in the Figure are, already from visual inspection, very similar to each other. This property produces a high dependence between layers, since the overlap is increased by the multiple presence of links in the various layers connecting nodes to the hubs.

It is possible to show that this hubs distribution, leading to the higher overlap between layers, is strongly correlated to the relation existing between the hidden variables *x_i_* associated to each node in the different layers. Indeed, as shown in Figure 4(c), for the considered pair of layers (but several pairs actually exhibit the same behaviour) such a trend can be clearly represented by a straight line, thus pointing out that nodes with higher *x_i_* in one layer (hence, with higher probability of establishing a link with any other node in that layer) generally also have higher *x_i_* in a different layer.

**Figure 5:**
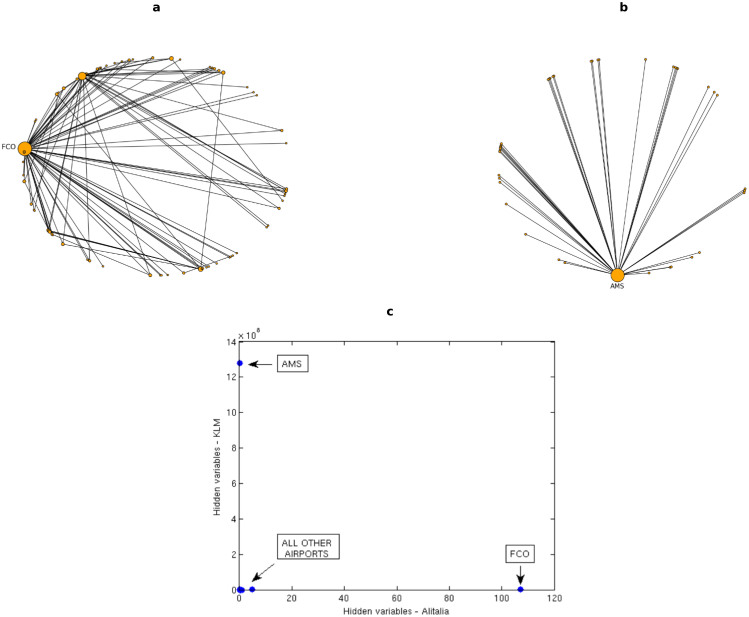
Hubs distribution in the European Airport multiplex. Top panels: graphs representing two layers of the system, respectively those associated to Alitalia airline (a) and KLM airline (b); nodes represent european airports; size of a node is proportional to its degree in that layer. All the observed links are reported. Bottom panel: scatter plot of the hidden variables *x_i_* relative to each of the nodes for the same two layers.

However, when the European Airport Network is considered, an opposite trend can be observed, thus a clear explanation of the small measured overlap applies; indeed, Figures 5(a) and 5(b) show that in this case the layers can be approximated to star-like graphs, with a single, largely connected hub and several other poorly connected nodes. Though, the hub is in general different for almost any considered layer, since each airline company is based on a different airport: in the considered pair of layers, hubs are represented by Rome - Fiumicino airport (FCO) for Alitalia and Amsterdam - Schiphol airport (AMS) for KLM. Such a property decreases significantly the overlap between layers, thus leading to the matrices previously shown in Figure 2.

Similar considerations can be done when looking at Figure 2(c), where the scatter plot of the hidden variables associated to the nodes in two different layers is shown. We observe that no linear trend can be inferred, since only the two hubs stand out from the bunch of the other airports (which are actually characterized by different values of *x_i_*, even though this cannot be fully appreciated). It is anyway clear that the hub of one layer, characterized by the highest value *x_i_* (hence, with the highest probability of establishing a link with any other node in that layer) is a poorly connected node in a different layer, being characterized by a small value of *x_i_*.

## Discussion

In the last few years the multiplex approach has revealed itself as a useful framework to study several real-world systems characterized by elementary units linked by different kinds of connection. In this context, we have introduced new measures aiming at analyzing dependencies between layers of the network, both for binary and weighted multi-graphs. We showed that our measures of multiplexity are able to extract crucial information from both sparse and dense networks by testing it on different real-world multi-layer systems. We clearly found that a distinction can be done based on the degree of overlap between links in different layers. For instance, we showed that some multiplexes exhibit small overlap between links in different layers, since just a limited number of nodes are active in many layers, while most of them participate to one or few layers. However, for other systems, such as the International Trade Network, most of the pairs of nodes are connected in several layers, so that such multiplexes exhibit large overlap between layers. Furthermore, we found that the multiplexity can also provide interesting information about the distribution of hubs across the various layers; indeed, systems characterized by nodes having many connections in most of the layers, such as the International Trade Network, tend to show higher values of raw binary multiplexity. On the other hand, in different networks, exhibiting values of multiplexity for most of the pairs of layers close to 0, a node with a low degree in a given layer may represent a hub in a different layer: the European Airport Network is a clear prototype of such systems.

Our findings suggest that adopting proper null models for multi-level networks, enforcing constraints taking into account dependencies between layers, is required in order to suitably model such real-world systems.

Further research in this direction will hopefully provide a better understanding of the role of local constraints in real-world multi-level systems.

## Methods

### Homogeneous null models

The simplest null model for a binary multiplex is an independent superposition of layers in which each layer is a Random Graph (RG)^28^, which enforces as constraint the expected number of links in that layer. Such model, therefore, provides a unique expected probability *p_α_* that a link between any two nodes is established in layer *α*: however, such a reference model completely discards any kind of heterogeneity in the degree distributions of the layers, resulting in graphs where each node has on average the same number of connections, inconsistently with the observed real networks. Thus, the probability of connection between any two nodes in layer *α* is uniformly given by:
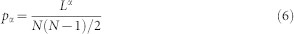
where *L^α^* is the total number of links actually observed in layer *α*.

Similar considerations apply to weighted networks and the related Weighted Random Graph (WRG)^25^, i.e. the straightforward extension of the previous Random Graph to weighted systems; in such a null model, the probability of having a link of weight *w* between two nodes *i* and *j* is independent from the choice of the nodes and only depends on the total weight observed in a layer and on the number of nodes.

Analogously to the corresponding Binary Random Graph, also this kind of null model discards the simultaneous presence of nodes with high and low values of the strengths (that is, by a high or low sum of the weights associated to links incident on that node).

### Heterogeneous null models

To take into account the heterogeneity of the real-world networks, in the unweighted case we consider a null model where the multiplex is an independent superposition of layers, each of which is a (Binary) Configuration Model (BCM)^32^, i.e. an ensemble of networks satisfying on average the empirical degree sequence observed in that specific layer. Since we make use of the canonical ensembles, it is possible to obtain from the Maximum Likelihood method each probability 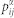 that nodes *i* and *j* are connected in layer *α* (notice that such value 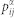 is basically the expectation value of 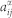 under the chosen Configuration Model). Similarly, as a null model for a weighted multiplex we consider an independent superposition of layers, each described by the Weighted Configuration Model (WCM)^33^: here, for each layer separately, the enforced constraint is the strength sequence as observed in the real-world multiplex. In this view, the likelihood maximization provides the expectation value of each weight 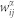 for any pair of nodes *i* and *j* as supplied by the Weighted Configuration Model. It is worth noticing that enforcing the degree sequence (respectively, the strength sequence in the weighted case) automatically leads to the design of a null model where also the total number of links (respectively, the total weight) of the network is preserved. In the Supplementary Information, we will provide equations generalizing, for instance, equation (6), whose solution allows then to derive the analytical expression of the expected link probability 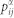 and, in the weighted case, the expected link weight 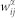. In order to do this, we make use of a set of *N* auxiliary variables 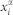 for any layer *α*, which are proportional to the probability of establishing a link between a given node *i* and any other node (or, respectively for the weighted case, establishing a link characterized by a given weight), being therefore directly informative on the expected probabilities 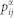 (or, respectively, the expected weights 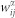).

## Author Contributions

V.G. analyzed the data and prepared the figures. D.G. planned the research. Both the authors wrote and reviewed the manuscript.

## Supplementary Material

Supplementary Information Multiplexity versus correlation: the role of local constraints in real multiplexes - Supplementary Information
