# Supplementary material for: Multiplexity versus correlation: the role of local constraints in real multiplexes
Source: Sci Rep. 2015 Mar 13;5:9120. doi: 10.1038/srep09120 (PMC4357874; doi:10.1038/srep09120)
Supplement: Supplementary Information — Multiplexity versus correlation: the role of local constraints in real multiplexes - Supplementary Information [file srep09120-s1.pdf]

Multiplexity versus correlation: the role of local constraints in real multiplexes - Supplementary Information -

V. Gemmetto[1,*] and D. Garlaschelli[1]

[1]Instituut-Lorentz for Theoretical Physics, Leiden Institute of Physics, University of Leiden, Niels Bohrweg 2, 2333 CA Leiden, The Netherlands

Abstract

Several systems can be represented as multiplex networks, i.e. in terms of a superposition of various graphs, each related to a different mode of connection between nodes. Hence, the definition of proper mathematical quantities aiming at capturing the added level of complexity of those systems is required. Various steps in this direction have been made. In the simplest case, dependencies between layers are measured via correlation-based metrics, a procedure that we show to be equivalent to the use of completely homogeneous benchmarks specifying only global constraints. However, this approach does not take into account the heterogeneity in the degree and strength distributions, which is instead a fundamental feature of real-world multiplexes. In this work, we compare the observed dependencies between layers with the expected values obtained from maximum-entropy reference models that appropriately control for the observed heterogeneity in the degree and strength distributions. This information-theoretic approach results in the introduction of novel and improved multiplexity measures that we test on different datasets, i.e. the International Trade Network and the European Airport Network. Our findings confirm that the use of homogeneous benchmarks can lead to misleading results, and highlight the important role played by the distribution of hubs across layers.

1 Uncorrelated null models for multi-layer networks

As in previous studies [1], we define the multiplex $\overrightarrow{G} = (G_1, G_2, \ldots, G_M)$ as the superposition of M layers G_k ($k = 1, 2, \ldots, M$), each of them represented by a (possibly weighted) network sharing the same set of N nodes with the other ones, although we do not require that all the vertices are active in each layer. Therefore, multiplex ensembles can be defined by associating a probability $P(\overrightarrow{G})$ to each multi-network, so that the entropy S of the ensemble is given by:

$$S = -\sum_{\overrightarrow{G}} P(\overrightarrow{G}) \ln P(\overrightarrow{G}) \tag{1}$$

It is then possible to design null models for multi-level networks by maximizing such an entropy after the enforcement of proper constraints. In this context, previous works [1, 2, 3] introduced the concepts of correlated and uncorrelated multiplex ensembles, based on the possibility to introduce correlations between layers within the null models. In particular, for an uncorrelated ensemble the probability of a given multiplex can be factorize into the probabilities of each single-layer network G_k belonging to that multiplex, as the links in any two layers α and β are uncorrelated; thus, it is given by:

$$P(\overrightarrow{G}) = \prod_{k=1}^{M} P_k(G_k) \tag{2}$$

Instead, if we want to take into account correlations between layers, the previous relation (2) does not hold.

As stated in the main text, as our purpose is precisely that of measuring such correlations, we are going to consider the former type of ensemble, in order to define a null model for the real system so that it is possible to compare the observed correlations with reference models where the overlap between layers is actually randomized and, at the same time, important properties of the real network are preserved.

In this perspective, therefore, the definition of proper null models for the considered multiplex reduces to the definition of an indipendent null model for any layer of the system. In order to do this, we take advantage of the concept of canonical network ensemble, or exponential random graph [4], i.e. the randomized family of graphs satisfying a set of constraints on average. In this context the resulting randomized graph preserves only part of the topology of the considered real-world network and is entirely random otherwise, thus it can be employed as a proper reference model.

However, fitting such previously defined models [1, 2, 3] to real datasets is hard, since it is usually computationally demanding as it requires the generation of many randomized networks whose properties of interest have to be measured. In this perspective, we make use of a fast and completely analytical Maximum Entropy method, based on the maximization of the likelihood function [5, 6, 7], which provides the exact probabilities of occurrence of random graphs with the same average constraints as the real network. From such probabilities it is then possible to compute the expectation values of the properties we are interested in, such as the average link probability or the average weight associated to the link established between any two nodes. This procedure is general enough to be applied to any network, including the denser ones, and does not require the sampling of the configuration space in order to compute average values of the quantities of interest. While the adoption of such a method is not strictly required when dealing with global constraints like the total number of links observed in a network, it becomes crucial when facing the problem of enforcing local constraints such as the degree sequence or the strength sequence.

Indeed, so far the most widely used graph null model has been represented by the Random Graph (RG) [4], which enforces on average as constraint the expected number of links in the network. Such model, therefore, provides a unique expected probability p_α that a link between any two nodes is established in layer α: however, as we said, such a reference model completely discards any kind of heterogeneity in the degree distributions of the layers, resulting in graphs where each node has on average the same number of connections, inconsistently

with the observed real networks. Thus, the probability of connection between any two nodes in layer α is uniformly given by:

$$p_\alpha = \frac{L^\alpha}{N(N-1)/2} \tag{3}$$

where L^α is the total number of links actually observed in layer α:

$$L^\alpha = \sum_{i<j} a_{ij}^\alpha \tag{4}$$

and $a_{ij}^\alpha = 0, 1$ depending on the presence of the link between nodes i and j in layer α.

Similar considerations apply to weighted networks and the related Weighted Random Graph (WRG) [8], i.e. the straightforward extension of the previous Random Graph to weighted systems; in such a null model, the probability of having a link of weight w between two nodes i and j is independent from the choice of the nodes, and it is given by the following geometric distribution:

$$P(w^\alpha) = {p_\alpha}^w (1 - p_\alpha) \tag{5}$$

where the Maximum Likelihood method shows that the optimal value of the parameter p_α is given by:

$$p_\alpha = \frac{2W^\alpha}{N(N-1) + 2W^\alpha} \tag{6}$$

with W^α defined as the total weight observed in layer α (w_{ij}^α is the weight associated to the link between nodes i and j in the same layer):

$$W^\alpha = \sum_{i<j} w_{ij}^\alpha \tag{7}$$

Similarly to the corresponding binary random graph, also this kind of null models discards the simultaneous presence of nodes characterized by high and low values of the strengths (that is, by a high or low sum of the weights associated to links incident on that node).

To take into account the heterogeneity of the real-world networks within the null models, in the unweighted case we consider the (Binary) Configuration Model (BCM) [9], i.e. the ensemble of networks satisfying on average a given degree sequence. Since we make use of the canonical ensembles, it is possible to obtain from the Maximum Likelihood method each probability p_{ij}^α that nodes i and j are connected in layer α (notice that such value p_{ij}^α is basically the expectation value of a_{ij}^α under the chosen Configuration Model). Similarly, for weighted graphs the Weighted Configuration Model (WCM) [10] can be defined: here, the enforced constraint is represented by the strength sequence as observed in the real-world network. In this view, the likelihood maximization provides the expectation value of each weight w_{ij}^α for any pair of nodes i and j as supplied by the Weighted Configuration Model. It is worth noticing that enforcing the degree sequence (respectively, the strength sequence in the weighted case) automatically leads to the design of a null model where also the total number of links (respectively, the total weight) of the network is preserved. In the following section, we will provide equations generalizing equations (3) and (6), whose solution allows then to derive the analytical expression of the expected link probability p_{ij}^α and, in the weighted case, the expected link weight w_{ij}^α. In order to do this, we make use of a set of N auxiliary variables x_i^α for any layer α, which are proportional to the probability of establishing a link between a given node i and any other node (or, respectively for the weighted case, establishing a link characterized by a given weight), being therefore directly informative on the expected probabilities p_{ij}^α (or, respectively, the expected weights w_{ij}^α).

2 Maximum Likelihood Method

We now briefly explain the Maximum Likelihood Method [6]. In the binary case, when the observed degree sequence represents the property that we want to preserve (i.e., in the so-called configuration model), the method reduces to finding the solution to following set of N coupled nonlinear equation, independently for each layer $\alpha = 1, 2, \ldots, M$:

$$\sum_{i<j} \frac{x_i^\alpha x_j^\alpha}{1 + x_i^\alpha x_j^\alpha} = k_i^\alpha \quad \forall i = 1, 2, \ldots, N \tag{8}$$

where k_i^α is the observed degree of node i in layer α and the unknown variables of the equation are the so-called N hidden variables associated to that layer.

Thus, the expected link probability p_{ij}^α is given by, for any pair of nodes (i, j) in any layer α:

$$p_{ij}^\alpha = \frac{x_i^\alpha x_j^\alpha}{1 + x_i^\alpha x_j^\alpha} \tag{9}$$

which is therefore the generalization of the expression (3) in the previous section. We can therefore see that such hidden variables x_i^α are proportional to the expected link probability p_{ij}^α in a given layer α: a higher value of x_i^α will correspond to a higher expected probability of observing a link between i and any other node $j \neq i$, and vice-versa.

Similarly, for weighted multiplexes, we can enforce the strength sequence observed in a real network on a network ensemble, thus designing a proper null model where the strength sequence of the considered real-world network is preserved, while the other properties are randomized. In this context, the Maximum Likelihood Method for weighted graphs reduces to solving a set of N coupled nonlinear equations. For any node i in any layer α, we have:

$$\sum_{i<j} \frac{x_i^\alpha x_j^\alpha}{1 - x_i^\alpha x_j^\alpha} = s_i^\alpha \tag{10}$$

where s_i^α is the observed strength of node i in layer α and the unknown variables of the equation are, again, the N hidden variables associated to the considered layer.

Thus, the expected link weight w_{ij}^α is given by, for any pair of nodes (i, j):

$$w_{ij}^\alpha = \frac{x_i^\alpha x_j^\alpha}{1 - x_i^\alpha x_j^\alpha} \tag{11}$$

hence generalizing the corresponding equation (6) of the main text. In this case, the computed hidden variables x_i^α are proportional to the expected link weight w_{ij}^α in a given layer α; a higher value of x_i^α will therefore correspond to a higher expected link weight between i and any other node $j \neq i$, and vice-versa.

We can now derive the expression for the expectation values of the binary and weighted multiplexity defined in the main text.

3 Binary multiplexity

When the unweighted networks are considered we have defined the "absolute" binary multiplexity between any two layers α and β as:

$$m_{bin}^{\alpha,\beta} = \frac{2 \sum_{i<j} \min\{a_{ij}^\alpha, a_{ij}^\beta\}}{L^\alpha + L^\beta} \tag{12}$$

with the previously introduced notation.

As we said, this quantity is informative only after a comparison with the value of binary multiplexity obtained when considering a null model. We have therefore introduced the following transformed or rescaled quantity [11, 12]:

$$\mu_{bin}^{\alpha,\beta} = \frac{m_{bin}^{\alpha,\beta} - \langle m_{bin}^{\alpha,\beta} \rangle}{1 - \langle m_{bin}^{\alpha,\beta} \rangle} \tag{13}$$

where $m_{bin}^{\alpha,\beta}$ is the value measured for the observed real-world multiplex and $\langle m_{bin}^{\alpha,\beta} \rangle$ is the value expected under the chosen null model. We will show in the next section that, when the Random Graph is considered as a null model, the previous quantity (13) is actually the correlation coefficient between the entries of the adjacency matrix referred to any two layers α and β of a multi-level graph.

We should point out that the raw intra-layer multiplexity $m_{bin}^{\alpha,\alpha}$ always leads to a measured value equal to 1, representing complete similarity between any layer and itself. However, the rescaled intra-layer multiplexity $\mu_{BCM}^{\alpha,\alpha}$ actually leads to an indeterminate value; therefore, we choose to set this value by construction equal to 1 too, for sake of clarity.

In order to compute $\mu_{bin}^{\alpha,\beta}$ we should then calculate the expected multiplexity under the chosen null model, that is:

$$\langle m_{bin}^{\alpha,\beta} \rangle = \frac{2\sum_{i<j} \langle \min\{a_{ij}^{\alpha}, a_{ij}^{\beta}\} \rangle}{\langle L^{\alpha} \rangle + \langle L^{\beta} \rangle} \tag{14}$$

However, since both the considered null models preserve the average number of links in each layer as constraint, we have just to evaluate the analytical expression for the expected value of the minimum of two variables. In the unweighted case, this is easy because it reduces to the evaluation of the expected minimum between two indipendent, binary variables. In particular, when the Configuration Model is considered (the extension to the Random Graph is straightforward), the probability that a link exists between nodes i and j is given by the mass probability function of a Bernoulli-distributed variable:

$$P(a_{ij}^{\alpha}) = p_{ij}^{a_{ij}} (1 - p_{ij})^{(1-a_{ij})} \tag{15}$$

Therefore, we have for the configuration model:

$$\begin{aligned} \langle \min\{a_{ij}^{\alpha}, a_{ij}^{\beta}\} \rangle_{BCM} &= \sum_{a_{ij}^{\alpha}, a_{ij}^{\beta}} \min\{a_{ij}^{\alpha}, a_{ij}^{\beta}\} P\left(\min\{a_{ij}^{\alpha}, a_{ij}^{\beta}\}\right) = \\ &= 0 \cdot P\left(\min\{a_{ij}^{\alpha}, a_{ij}^{\beta}\} = 0\right) + 1 \cdot P\left(\min\{a_{ij}^{\alpha}, a_{ij}^{\beta}\} = 1\right) = \\ &= P\left(\min\{a_{ij}^{\alpha}, a_{ij}^{\beta}\} = 1\right) = \\ &= P\left(a_{ij}^{\alpha} = 1\right) P\left(a_{ij}^{\beta} = 1\right) = \\ &= p_{ij}^{\alpha} p_{ij}^{\beta} \end{aligned} \tag{16}$$

and similarly for the Random Graph:

$$\langle \min\{a_{ij}^{\alpha}, a_{ij}^{\beta}\} \rangle_{RG} = p^{\alpha} p^{\beta} \tag{17}$$

where we define p^{α} as the fraction of links actually present in that layer, as we have already done before:

$$p^{\alpha} = \frac{L^{\alpha}}{N(N-1)/2} \tag{18}$$

It is now possible to compute the analytical expression for the rescaled multiplexity. We obtain for the Random Graph:

$$\mu_{RG}^{\alpha,\beta} = \frac{2\sum_{i<j}\left(\min\{a_{ij}^{\alpha}, a_{ij}^{\beta}\} - p^{\alpha}p^{\beta}\right)}{\sum_{i<j}\left(a_{ij}^{\alpha} + a_{ij}^{\beta} - 2p^{\alpha}p^{\beta}\right)} \tag{19}$$

and for the Binary Configuration Model:

$$\mu_{BCM}^{\alpha,\beta} = \frac{2\sum_{i<j}\left(\min\{a_{ij}^{\alpha}, a_{ij}^{\beta}\} - p_{ij}^{\alpha}p_{ij}^{\beta}\right)}{\sum_{i<j}\left(a_{ij}^{\alpha} + a_{ij}^{\beta} - 2p_{ij}^{\alpha}p_{ij}^{\beta}\right)} \tag{20}$$

3.1 Binary multiplexity: z-scores

As we have already said, such rescaled quantities provide proper information about the similarity between layers of a multiplex, by evaluating the dependencies measured in a real network with respect to what we would expect, on average, for an ensemble of multi-level networks sharing only some of the topological properties of the observed one. However, we cannot understand, from the obtained values of multiplexity itself, whether the observed value of m_{bin} is actually compatible with the expected one, as μ_{BCM} (and the correspondig value related to the Random Graph) does not provide any information about the standard deviation associated to the expected value of multiplexity.

In order to solve this issue, we introduce the z-score associated to the previously defined multiplexity:

$$z\left[m^{\alpha,\beta}\right] = \frac{m^{\alpha,\beta} - \langle m^{\alpha,\beta}\rangle}{\sigma\left[m^{\alpha,\beta}\right]} \tag{21}$$

where $m^{\alpha,\beta}$ is the measured multiplexity between a given pair of layers on the real-world network, $\langle m^{\alpha,\beta}\rangle$ is the value expected under the chosen null model and $\sigma[m^{\alpha,\beta}]$ is the related standard deviation. The z-score, therefore, shows by how many standard deviations the observed value of multiplexity differs with respect to the expected one for any pair of layers. In particular, in the binary case such a quantity becomes:

$$z\left[m^{\alpha,\beta}\right] = \frac{\sum_{i<j} min\{a_{ij}^{\alpha}, a_{ij}^{\beta}\} - \sum_{i<j}\langle min\{a_{ij}^{\alpha}, a_{ij}^{\beta}\}\rangle}{\sigma\left[\sum_{i<j} min\{a_{ij}^{\alpha}, a_{ij}^{\beta}\}\right]} \tag{22}$$

Interestingly, not only the expected value, but even the standard deviation can be calculated analytically. Indeed:

$$\sigma^2\left[\min\{a_{ij}^{\alpha}, a_{ij}^{\beta}\}\right] = \langle \min^2\{a_{ij}^{\alpha}, a_{ij}^{\beta}\}\rangle - \langle \min\{a_{ij}^{\alpha}, a_{ij}^{\beta}\}\rangle^2 \tag{23}$$

Exploiting again the binary character of the two indipendent variables a_{ij}^{α} and a_{ij}^{β}, the expected value of the square of the minimum becomes for the Configuration Model:

$$\begin{aligned}
\langle \min^2\{a_{ij}^{\alpha}, a_{ij}^{\beta}\}\rangle_{BCM} &= \sum_{a_{ij}^{\alpha}, a_{ij}^{\beta}} \min^2\{a_{ij}^{\alpha}, a_{ij}^{\beta}\} P\left(\min\{a_{ij}^{\alpha}, a_{ij}^{\beta}\}\right) = \\
&= 0\cdot P\left(\min\{a_{ij}^{\alpha}, a_{ij}^{\beta}\} = 0\right) + 1\cdot P\left(\min\{a_{ij}^{\alpha}, a_{ij}^{\beta}\} = 1\right) = \\
&= P\left(\min\{a_{ij}^{\alpha}, a_{ij}^{\beta}\} = 1\right) = \\
&= P\left(a_{ij}^{\alpha} = 1\right) P\left(a_{ij}^{\beta} = 1\right) = \\
&= p_{ij}^{\alpha}p_{ij}^{\beta}
\end{aligned} \tag{24}$$

Therefore, the standard deviation, required in order to evaluate the z-score associated to the multiplexity, is given by:

$$\sigma\left[\sum_{i<j}\min\{a_{ij}^{\alpha},a_{ij}^{\beta}\}\right]=\sqrt{\sum_{i<j}\left[p_{ij}^{\alpha}p_{ij}^{\beta}-\left(p_{ij}^{\alpha}p_{ij}^{\beta}\right)^{2}\right]} \tag{25}$$

The analytical value of the z-score related to the binary multiplexity, when the Configuration Model is taken into account, is then:

$$z_{BCM}=\frac{\sum_{i<j}\min\{a_{ij}^{\alpha},a_{ij}^{\beta}\}-\sum_{i<j}p_{ij}^{\alpha}p_{ij}^{\beta}}{\sqrt{\sum_{i<j}\left[p_{ij}^{\alpha}p_{ij}^{\beta}-\left(p_{ij}^{\alpha}p_{ij}^{\beta}\right)^{2}\right]}} \tag{26}$$

Extending such results to the Random Graph is immediate, since everything reduces to a change in the definition of the probability of observing a link between any given pair of nodes in each layer. Hence, the z-score associated to the binary multiplexity according to the binary Random Graph is given by:

$$z_{RG}=\frac{\sum_{i<j}\min\{a_{ij}^{\alpha},a_{ij}^{\beta}\}-\sum_{i<j}p^{\alpha}p^{\beta}}{\sqrt{\sum_{i<j}\left[p^{\alpha}p^{\beta}-(p^{\alpha}p^{\beta})^{2}\right]}} \tag{27}$$

where we used the previous definitions for p^{α} and p^{β}.

We should point out that such z-scores should in principle be defined only if the associated property (in this case, μ_{BCM}) is normally distributed; nevertheless, even if such assumption does not occur, they provide important information about the consistency between observed and randomized values. It is worth saying that these z-scores provide a different kind of information with respect to the previous multiplexities. Mathematically, the only correlation between, for example, μ_{BCM} and the corresponding z_{BCM} is the sign concordance; furthermore, the z-score is useful in order to understand whether, for instance, values of multiplexity close to 0 are actually comparable with 0, so that we can consider those two layers as uncorrelated, or they are instead significantly unexpected, although very small. In this perspective, we should not expect a particular relation between such two variables μ_{BCM} and z_{BCM} (or, respectively, μ_{RG} and z_{RG}).

3.2 Relationship with the correlation coefficient

A possible definition [13] of correlation between layers of a multiplex builds on the standard correlation coefficient:

$$Corr\{a_{ij}^{\alpha},a_{ij}^{\beta}\}=\frac{\langle a_{ij}^{\alpha}a_{ij}^{\beta}\rangle-\langle a_{ij}^{\alpha}\rangle\langle a_{ij}^{\beta}\rangle}{\sigma_{\alpha}\sigma_{\beta}} \tag{28}$$

Hence, a value of correlation equal to 0 represents a pair of uncorrelated layers only if the probability distributions of a_{ij}^{α} and a_{ij}^{β} are independent from the chosen node, that is, if all the edges in a certain layer are statistically equivalent. However, this leads to a probability of establishing a given link which is common to each pair of nodes, and this is the assumption behind the Random Graph.

In this context, it is then possible to show that, when the Binary Random Graph is taken into consideration, our novel measure of multiplexity can be reduced to the usual definition of correlation coefficient. Indeed, we

have:

$$\begin{aligned}\langle a^{\alpha}_{ij}a^{\beta}_{ij}\rangle &= \frac{2\sum_{i<j} a^{\alpha}_{ij}a^{\beta}_{ij}}{N(N-1)} = \\ &= \frac{2\sum_{i<j}\min\{a^{\alpha}_{ij}, a^{\beta}_{ij}\}}{L^{\alpha}+L^{\beta}}\frac{L^{\alpha}+L^{\beta}}{N(N-1)} = \\ &= m^{\alpha,\beta}\frac{L^{\alpha}+L^{\beta}}{N(N-1)} \end{aligned} \tag{29}$$

Moreover, the average value of a^{α}_{ij} over all the pairs of nodes in layer α is given by:

$$\langle a^{\alpha}_{ij}\rangle = \frac{2L^{\alpha}}{N(N-1)} \tag{30}$$

and similarly for layer β:

$$\langle a^{\beta}_{ij}\rangle = \frac{2L^{\beta}}{N(N-1)} \tag{31}$$

Hence,

$$\langle a^{\alpha}_{ij}\rangle\langle a^{\beta}_{ij}\rangle = \frac{4L^{\alpha}L^{\beta}}{N^2(N-1)^2} \tag{32}$$

On the contrary, the expected value of multiplexity under random graph is given by:

$$\begin{aligned}\langle m^{\alpha,\beta}\rangle &= \frac{2\sum_{i<j} p^{\alpha}p^{\beta}}{L^{\alpha}+L^{\beta}} = \\ &= \frac{N(N-1)}{L^{\alpha}+L^{\beta}}\frac{2L^{\alpha}}{N(N-1)}\frac{2L^{\beta}}{N(N-1)} = \\ &= \frac{1}{N(N-1)}\frac{4L^{\alpha}L^{\beta}}{L^{\alpha}+L^{\beta}} \end{aligned} \tag{33}$$

There is therefore a direct relation between $\langle a^{\alpha}_{ij}\rangle\langle a^{\beta}_{ij}\rangle$ and $\langle m^{\alpha,\beta}\rangle$:

$$\begin{aligned}\langle a^{\alpha}_{ij}\rangle\langle a^{\beta}_{ij}\rangle &= \frac{4L^{\alpha}L^{\beta}}{N^2(N-1)^2} = \\ &= \langle m^{\alpha,\beta}\rangle\frac{L^{\alpha}+L^{\beta}}{N(N-1)} \end{aligned} \tag{34}$$

Furthermore, we need to derive the expression for the standard deviation σ_{α} and σ_{β}:

$$\begin{aligned}\sigma_{\alpha} &= \sqrt{\langle\left(a^{\alpha}_{ij}\right)^2\rangle - \langle a^{\alpha}_{ij}\rangle^2} = \\ &= \sqrt{\langle a^{\alpha}_{ij}\rangle\left(1-\langle a^{\alpha}_{ij}\rangle\right)} = \\ &= \sqrt{\frac{2L^{\alpha}}{N(N-1)}\left[1-\frac{2L^{\alpha}}{N(N-1)}\right]} \end{aligned} \tag{35}$$

and analogously for β. Hence, the correlation coefficient between a_{ij}^{α} and a_{ij}^{β} is given by:

$$\begin{aligned} Corr\{a_{ij}^{\alpha}, a_{ij}^{\beta}\} &= \frac{\frac{L^{\alpha}+L^{\beta}}{N(N-1)} m^{\alpha,\beta} - \frac{L^{\alpha}+L^{\beta}}{N(N-1)} \langle m^{\alpha,\beta} \rangle}{\frac{2}{N(N-1)} \sqrt{L^{\alpha} L^{\beta} \left(1 - \frac{2L^{\alpha}}{N(N-1)}\right) \left(1 - \frac{2L^{\beta}}{N(N-1)}\right)}} \\ &= \frac{\left(L^{\alpha} + L^{\beta}\right) \left(m^{\alpha,\beta} - \langle m^{\alpha,\beta} \rangle\right)}{2\sqrt{L^{\alpha} L^{\beta} \left(1 - \frac{2L^{\alpha}}{N(N-1)}\right) \left(1 - \frac{2L^{\beta}}{N(N-1)}\right)}} \end{aligned} \quad (36)$$

It is therefore clear that, apart from a different normalization factor (depending on L^{α} and L^{β}), our definition of binary rescaled multiplexity, when the Random Graph is considered as null model, reduces to the usual correlation coefficient (28).

However, such a property does not hold when a different reference model, such as the Configuration Model, is considered.

4 Weighted multiplexity

In the main text, we have also extended the previous definitions to weighted multiplex networks. We have defined the "absolute" weighted multiplexity as:

$$m_{w}^{\alpha,\beta} = \frac{2\sum_{i<j} min\{w_{ij}^{\alpha}, w_{ij}^{\beta}\}}{W^{\alpha} + W^{\beta}} \quad (37)$$

where w_{ij}^{α} represents the weight of the link between nodes i and j in layer α and W^{α} is the total weight related to the links in that layer.

Furthermore, we have defined the following transformed or rescaled quantity:

$$\mu_{w}^{\alpha,\beta} = \frac{m_{w}^{\alpha,\beta} - \langle m_{w}^{\alpha,\beta} \rangle}{1 - \langle m_{w}^{\alpha,\beta} \rangle} \quad (38)$$

where $\langle m_{w}^{\alpha,\beta} \rangle$ is the value measured for the observed real-world network and $\langle m_{w}^{\alpha,\beta} \rangle$ is the value expected under the considered reference model. Again, the sign of $\mu_{w}^{\alpha,\beta}$ is then directly informative about the weighted dependency existing between layers.

In this context, the expected value of weighted multiplexity is given by:

$$\langle m_{w}^{\alpha,\beta} \rangle = \frac{2\sum_{i<j} \langle \min\{w_{ij}^{\alpha}, w_{ij}^{\beta}\} \rangle}{\langle W^{\alpha} \rangle + \langle W^{\beta} \rangle} \quad (39)$$

However, since both the Weighted Random Graph and the Weighted Configuration Model preserve the average total weight associated to the links in each layer as constraint, also in this case we just need to evaluate the analytical expression for the expected value of the minimum of two variables; the only difference with respect to the binary description is related to a change in the underlying probability distribution [11].

Indeed, in the weighted case, when the Weighted Configuration Model is considered (again, the extension to the Weighted Random Graph is straightforward) such variables are distributed according to a geometrical distribution:

$$P(w_{ij}^{\alpha}) = p_{ij}^{w_{ij}^{\alpha}} (1 - p_{ij}^{\alpha}) \quad (40)$$

In order to quantify such an expectation value, we exploit the cumulative distribution of the minimum between the considered variables:

$$\begin{aligned} P\left(\min\{w_{ij}^{\alpha}, w_{ij}^{\beta}\} \geq w\right) &= P\left(w_{ij}^{\alpha} \geq w\right) P\left(w_{ij}^{\beta} \geq w\right) = \\ &= \left(p_{ij}^{\alpha} p_{ij}^{\beta}\right)^{w} \end{aligned} \tag{41}$$

Thus, the expected minimum, under Weighted Configuration Model, becomes:

$$\begin{aligned} \langle \min\{w_{ij}^{\alpha}, w_{ij}^{\beta}\} \rangle_{WCM} &= \sum_{w'} w' [P\left(\min\{w_{ij}^{\alpha}, w_{ij}^{\beta}\} \geq w'\right) - P\left(\min\{w_{ij}^{\alpha}, w_{ij}^{\beta}\} \geq w' + 1\right)] = \\ &= \sum_{w'} w' \left[\left(p_{ij}^{\alpha} p_{ij}^{\beta}\right)^{w'} - \left(p_{ij}^{\alpha} p_{ij}^{\beta}\right)^{w'+1}\right] = \\ &= \frac{p_{ij}^{\alpha} p_{ij}^{\beta}}{1 - p_{ij}^{\alpha} p_{ij}^{\beta}} \end{aligned} \tag{42}$$

and, for the Weighted Random Graph:

$$\langle \min\{w_{ij}^{\alpha}, w_{ij}^{\beta}\} \rangle_{WRG} = \frac{p^{\alpha} p^{\beta}}{1 - p^{\alpha} p^{\beta}} \tag{43}$$

where we define p^{α}, according to the likelihood maximization, as:

$$p^{\alpha} = \frac{W^{\alpha}}{W^{\alpha} + N(N-1)/2}, \tag{44}$$

We can now compute the analytical expression for the rescaled multiplexity, according to both the chosen null models. We obtain for the Weighted Random Graph:

$$\mu_{WRG}^{\alpha,\beta} = \frac{2 \sum_{i<j} \left(\min\{w_{ij}^{\alpha}, w_{ij}^{\beta}\} - \frac{p^{\alpha} p^{\beta}}{1 - p^{\alpha} p^{\beta}}\right)}{\sum_{i<j} \left(w_{ij}^{\alpha} + w_{ij}^{\beta} - 2 \frac{p^{\alpha} p^{\beta}}{1 - p^{\alpha} p^{\beta}}\right)} \tag{45}$$

and for the Weighted Configuration Model:

$$\mu_{WCM}^{\alpha,\beta} = \frac{2 \sum_{i<j} \left(\min\{w_{ij}^{\alpha}, w_{ij}^{\beta}\} - \frac{p_{ij}^{\alpha} p_{ij}^{\beta}}{1 - p_{ij}^{\alpha} p_{ij}^{\beta}}\right)}{\sum_{i<j} \left(w_{ij}^{\alpha} + w_{ij}^{\beta} - 2 \frac{p_{ij}^{\alpha} p_{ij}^{\beta}}{1 - p_{ij}^{\alpha} p_{ij}^{\beta}}\right)} \tag{46}$$

with the previously defined notation.

4.1 Weighted multiplexity: z-scores

Furthermore, we can extend to the weighted case the analysis of the z-scores associated to the values of multiplexity as defined in (38). We can define it in the usual way:

$$z\left[m^{\alpha,\beta}\right] = \frac{\sum_{i<j} \min\{w_{ij}^{\alpha}, w_{ij}^{\beta}\} - \sum_{i<j} \langle \min\{w_{ij}^{\alpha}, w_{ij}^{\beta}\} \rangle}{\sigma\left[\sum_{i<j} \min\{w_{ij}^{\alpha}, w_{ij}^{\beta}\}\right]} \tag{47}$$

Since:

$$\sigma^2\left[\min\{w_{ij}^{\alpha},w_{ij}^{\beta}\}\right]=\langle\min^2\{w_{ij}^{\alpha},w_{ij}^{\beta}\}\rangle-\langle\min\{w_{ij}^{\alpha},w_{ij}^{\beta}\}\rangle^2 \tag{48}$$

we just have to compute the analytical expression for the expected value of the square of minimum bewteeen w_{ij}^{α} and w_{ij}^{β}. Then, following the same procedure adopted for (42) we find:

$$\begin{aligned}\langle\min^2\{w_{ij}^{\alpha},w_{ij}^{\beta}\}\rangle_{WCM} &= \sum_{w'}(w')^2\left[P\left(\min\{w_{ij}^{\alpha},w_{ij}^{\beta}\}\geq w'\right)-P\left(\min\{w_{ij}^{\alpha},w_{ij}^{\beta}\}\geq w'+1\right)\right]=\\ &=\sum_{w'}(w')^2\left[\left(p_{ij}^{\alpha}p_{ij}^{\beta}\right)^{w'}-\left(p_{ij}^{\alpha}p_{ij}^{\beta}\right)^{w'+1}\right]=\\ &=\frac{p_{ij}^{\alpha}p_{ij}^{\beta}+\left(p_{ij}^{\alpha}p_{ij}^{\beta}\right)^2}{\left(1-p_{ij}^{\alpha}p_{ij}^{\beta}\right)^2}\end{aligned} \tag{49}$$

and therefore the standard deviation is:

$$\sigma\left[\sum_{i<j}\min\{w_{ij}^{\alpha},w_{ij}^{\beta}\}\right]=\sqrt{\sum_{i<j}\left[\frac{p_{ij}^{\alpha}p_{ij}^{\beta}+\left(p_{ij}^{\alpha}p_{ij}^{\beta}\right)^2}{\left(1-p_{ij}^{\alpha}p_{ij}^{\beta}\right)^2}-\frac{\left(p_{ij}^{\alpha}p_{ij}^{\beta}\right)^2}{\left(1-p_{ij}^{\alpha}p_{ij}^{\beta}\right)^2}\right]} \tag{50}$$

Finally, the z-score associated to the weighted multiplexity under Weighted Configuration Model is therefore given by:

$$z_{WCM}=\frac{\sum_{i<j}\min\{w_{ij}^{\alpha},w_{ij}^{\beta}\}-\sum_{i<j}\frac{p_{ij}^{\alpha}p_{ij}^{\beta}}{1-p_{ij}^{\alpha}p_{ij}^{\beta}}}{\sqrt{\sum_{i<j}\frac{p_{ij}^{\alpha}p_{ij}^{\beta}}{\left(1-p_{ij}^{\alpha}p_{ij}^{\beta}\right)^2}}} \tag{51}$$

Analogously, we get:

$$z_{WRG}=\frac{\sum_{i<j}\min\{w_{ij}^{\alpha},w_{ij}^{\beta}\}-\sum_{i<j}\frac{p^{\alpha}p^{\beta}}{1-p^{\alpha}p^{\beta}}}{\sqrt{\sum_{i<j}\frac{p^{\alpha}p^{\beta}}{(1-p^{\alpha}p^{\beta})^2}}} \tag{52}$$

for the Weighted Random Graph, where we used the previous definitions for p^{α} and p^{β}.

5 Additional results

As we stated in the main text, in order to have a better understanding of the correlations between layers, it is possible to implement a hierarchical clustering procedure starting from each of the multiplexity matrices shown in the main text [14]. However, we have to define a notion of distance between layers, starting from our notion of dependency. We can define a distance $d^{\alpha,\beta}$ between any pair of commodities in the following way:

$$d^{\alpha,\beta}=\sqrt{\frac{1-\mu_{BCM}^{\alpha,\beta}}{2}}. \tag{53}$$

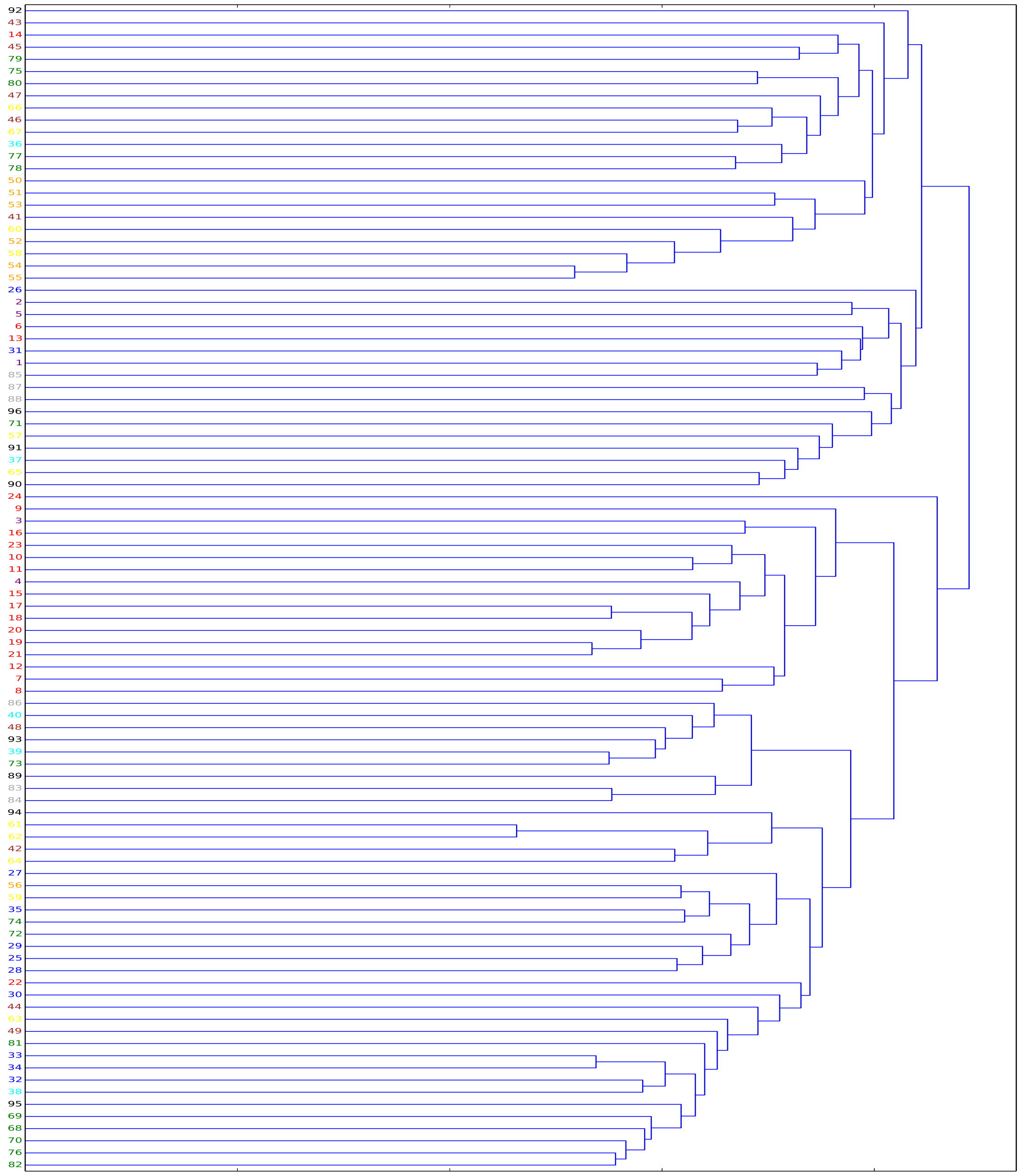

Figure 1: Dendrogram of commodities traded in 2011 as obtained applying the Average Linkage Clustering Algorithm to the binary rescaled multiplexity μ_{BCM}; colors of the leaves represent different classes of commodities, as reported in the last Section of the Supplementary Information.

where we chose to consider, for instance, the transformed multiplexity under Binary Configuration Model. Hence, the maximum possible distance $d^{\alpha,\beta}$ between any two layers is 1 (when layers α and β show multiplexity $\mu^{\alpha,\beta}_{BCM} = 1$), while the minimum one is 0 (corresponding to $\mu^{\alpha,\beta}_{BCM} = -1$). We can therefore represent the layers of the multiplex as the leaves of a taxonomic tree, where highly correlated communities meet at a branching point which is closer to baseline level. In Figure 1 we show the dendrogram obtained by applying the Average Linkage Clustering Algorithm to the matrix representing values of multiplexity μ_{BCM}. We can see that some groups of similar commodities are clearly visible (for instance, the group of edible commodities can be easily identified), while in other cases apparently distant commodities are grouped together, pointing out that some unexpected dependencies are present. The dendrogram reported in Figure 1 therefore represents a refinement of the taxonomic tree reported in [13]. Similar dendrograms can be designed starting from the matrices representing values of μ_{RG} or weighted multiplexity μ_{WRG} and μ_{WCM}.

Moreover, it is possible to perform the same analysis on the European Airport Network. However, a dendrogram in this case would not be meaningful, since most of the layers meet at a single root level, due to the very low correlation observed between them.

As we said, color-coded multiplexity matrices, as shown in the main text, are useful in order to detect the meaninful dependencies between layers in a multiplex, but they do not supply any information about the discrepancy of the observed values from the corresponding expected ones. Hence, the introduction of suitable z-scores associated to the previously defined quantities is required. Moreover, it is worth reminding that the information provided by (20) (respectively (19) for the Random Graph) is not necessarily connected to that supplied by (26) (respectively, (27)) Indeed, while the multiplexity by itself detects the degree of correlation between layers of a multi-level network, the corresponding z-scores reveal how significant those values actually are with respect to our expectations. In Figure 2(a) we show, for the International Trade Network, the scatter

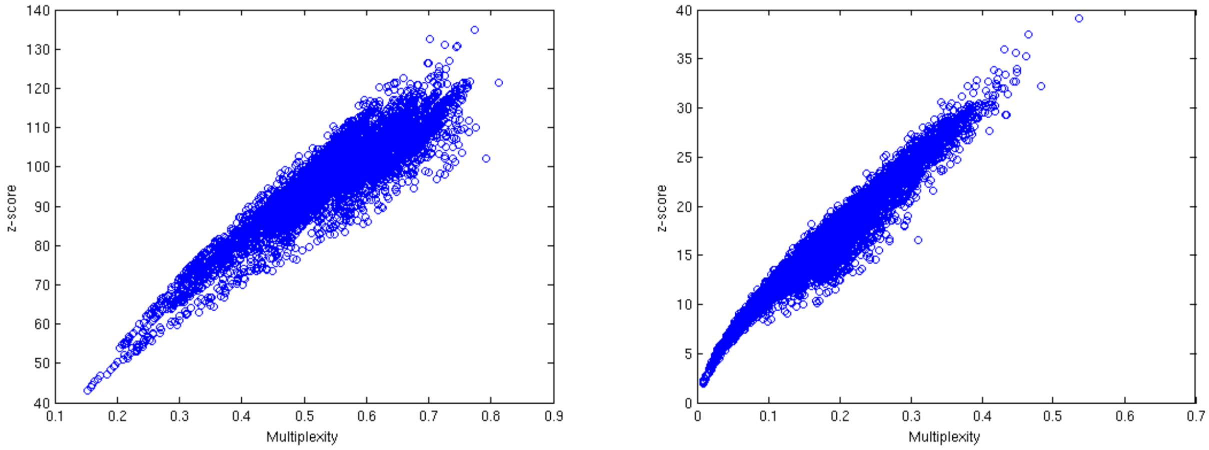

Figure 2: Scatter plots of binary multiplexity values $\mu^{\alpha,\beta}_{bin}$ vs the corresponding z-score for each pair of layers, respectively for Random Graph (a) and Binary Configuration Model (b), for the International Trade Network.

plot of the values of binary multiplexity versus the corresponding z-scores, after comparing the observed values with the expected ones under Random Graph. We show that observed very large values of z-scores reveal a high significance of the previously obtained overlaps; such a consideration therefore points out that even the pairs of layers showing low (but positive) values of multiplexity cannot actually be considered as uncorrelated. Furthermore, a clear correlation between μ_{RG} and z_{RG} can be observed, thus large values of binary multiplexity correspond to large z-scores, and vice-versa.

Similar considerations can be done when the Binary Configuration Model is considered as a beachmark.

Indeed, as we show in Figure 2(b), a large correlation between μ_{BCM} and z_{BCM} is still present when we consider the International Trade Network; moreover, since almost all the z-scores are higher than the widely used critical value $z^*_{BCM} = 2$ (so that almost no pair of layers shows a multiplexity lying within 2 standard deviations form the expected value), we highlight that most of the pairs therefore exhibit unexpectedly high correlations with respect to the corresponding average value obtained when randomizing the real-world layers according to the Configuration Model, similarly to what we found before for the Random Graph.

However, if we look at the absolute values of such z-scores, we observe that the significance of the values of multiplexity under Random Graph (μ_{RG}) is generally much higher than that measured under Binary Configuration Model (μ_{BCM}). This property, which will still be true in the following Figures, is actually not surprising, since the Configuration Model enforces more constraints and therefore leads to higher similarity with the real network w.r.t the Random Graph.

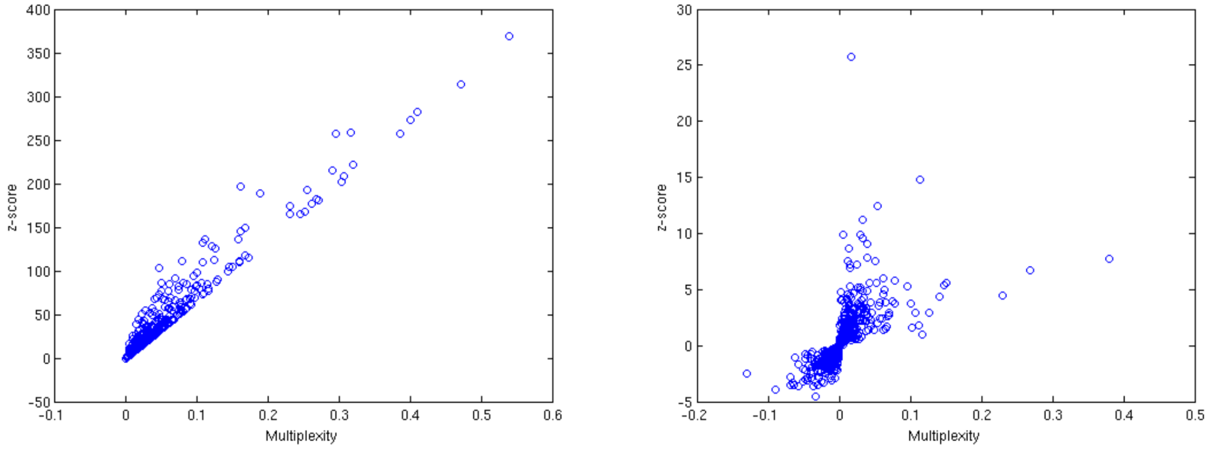

Figure 3: Scatter plots of binary multiplexity values $\mu^{\alpha,\beta}_{bin}$ vs the corresponding z-score for each pair of layers, respectively for Random Graph (a) and Binary Configuration Model (b), for the European Airport Network.

A different trend can be observed when the European Airport Network is taken into account (Figure 3(a)). Indeed, it is still clear a high correlation between values of multiplexity and their respective z-scores when the Random Graph is considered. However, many z-scores associated to multiplexities close to 0, in this case, are now close to 0 themselves, therefore suggesting that many pairs of layers (i.e. airline companies) may actually be anti-correlated rather simply uncorrelated. In this case, the adoption of a more refined null model is then crucial in order to deeply understand the structural properties of such a system.

When the Binary Configuration Model is considered as benchmark, however, the analysis of the corresponding scatter plots dramatically changes. However, as we said, these results are strongly dependent on the considered network. Indeed, Figure 3(b) exhibits a completely different trend with respect, for instance, to the corresponding Figure 2(b) (related to the World Trade Network): no correlation between μ_{BCM} and z_{BCM} can be observed in this case, so that the same value of multiplexity can be either associated to a low z-score (thus being compatible with the expected value under the chosen Configuration Model) or to very high z-scores (hence unexpectedly different from the model's expectation). Moreover, Figure 3(b) clearly shows the sign-concordance existing between the multiplexity and the associated z-score that we pointed out in the previous Section. However, no other clear trend can be inferred from such a plot, therefore pointing out the importance of taking into account both the quantities (μ_{BCM} and z_{BCM}) in order to have a complete understanding of the correlations between layers of a multiplex.

Furthermore, we should highlight once more that, in terms of absolute z-scores values, the significance of

the values of multiplexity under Random Graph (μ_{RG}) is usually much higher than that observed after the comparison with the Configuration Model (μ_{BCM}), as we have already found before for the International Trade Network.

Similarly, we can analyze the patterns of correlations resulting from the z-scores associated to the weighted multiplexity, as defined in (52) and (51). In Figure 4(a) we show the relation between the values of weighted multiplexity for any pair of layers and the related z-score, computed with respected to the expected multiplexity according to the Weighted Random Graph. The sign concordance is still clear, but the correlation between μ_{WRG} and z_{WRG} is much less sharp with respect to the corresponding binary case, especially for negative values of multiplexity.

Even more so, such a weak correlation between weighted multiplexity and the corresponding z-score completely disappears when the considered benchmark is the Weighted Configuration Model (Figure 4(b)): in this case the same value of μ_{WCM} may correspond to z-scores even characterized by different orders of magnitude, thus pointing out once more the importance of the introduction of a notion of standard deviation referred to the average $\langle \mu_{WCM} \rangle$. Indeed, the same value of observed multiplexity can actually be either extremely unexpected or in full agreement with the null model's prediction.

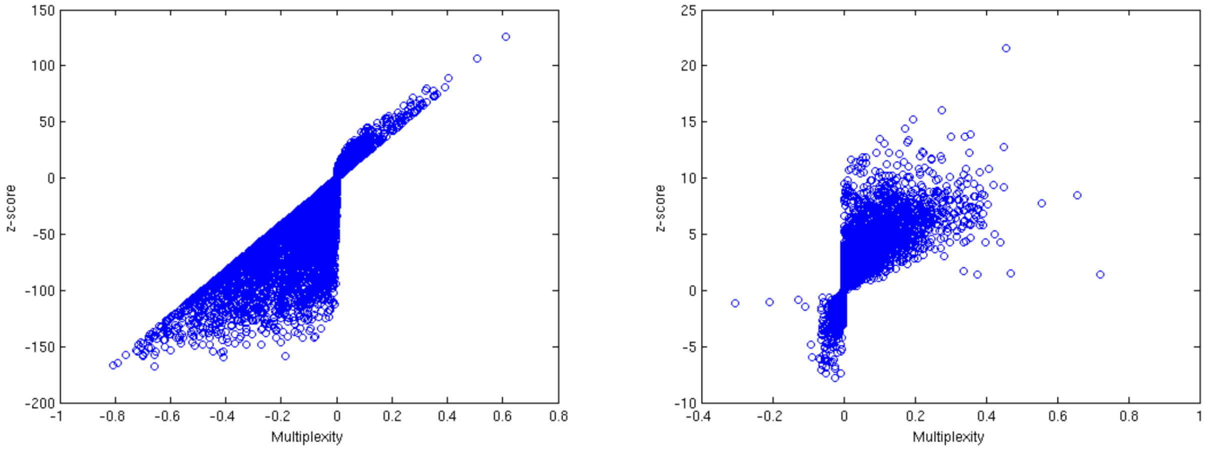

Figure 4: Scatter plots of weighted multiplexity values $\mu_w^{\alpha,\beta}$vs the corresponding z-score for each pair of layers, respectively for Weighted Random Graph (a) and Weighted Configuration Model (b), for the International Trade Network.

6 International Trade Network and European Airport Network: list of layers

We have analyzed the International Trade Network, also known as World Trade Web, as provided by the BACI database [15]. The data provide information about import and export between $N = 207$ countries in 2011 and turns out to have a straightforward representation in terms of multi-layered network [13]; it is indeed possible to disaggregate the global trade between any two countries into the import and export in a given commodity, so that the global trade system can be thought as the superposition of all the layers. The network is then composed by 207 countries and $M = 96$ different commodities, according to the standard international classification HS1996 [16] (the list of commodities is reported in Table 1). While the aggregated network shows a density higher than 55%, the various layers are characterized by densities from 6% (related to trade in silk)

to 45% (for import-export of mechanical appliances and parts thereof). Such heterogeneity may suggest that a multiplex analysis is therefore required. Interestingly, in this case each of the layers is represented by a weighted network, where the weight associated to any link in a layer stands for the amount of money exchanged by a given pair of countries in that layer (i.e., commodity).

The second multi-level network we have analyzed is the European Airport System. Here, the european airports represent the nodes, and the layer stand for the different European-based airline companies; hence, in a given layer a link between two nodes is present if there exists at least one direct flight between those two airports, operated by that airline. The dataset we consider has been provided by OpenFlight [17], a free on-line platform supplying information about the flights taking place all over the world. As we said before, we focus on the european network: $N = 669$ airports are thus considered, reached by flights operated by $M = 130$ companies (listed in Table 2). Unlike the International Trade Network, such a system can only be studied from an unweighted perspective, according to the chosen dataset. Moreover, both the aggregated network and the various layers show densities which are significantly lower than those observed in the previous dataset: indeed, in this system the aggregated network exhibits a density of about 2%, since a given airport has a number of connections with other cities which is very limited with respect to the global number of european airports.

We must therefore point out once more that the introduction of proper null models and the use of rescaled quantities allow us to appropriately compare the results obtained for such different networks, independently from the size and the density of the considered systems.

Code	Commodity	Class
01	Live animals	●
02	Meat and edible meat offal	●
03	Fish, crustaceans and acquatic invertebrates	●
04	Dairy produce; birs eggs; honey and other edible animal products	●
05	Other products of animal origin	●
06	Live trees, plants; bulbs, roots; cut flowers and ornamental foliage tea and spices	●
07	Edible vegetables and certain roots and tubers	●
08	Edible fruit and nuts; citrus fruit or melon peel	●
09	Coffee, tea, mate and spices	●
10	Cereals	●
11	Milling products; malt; starch; inulin; wheat gluten	●
12	Oil seeds and oleaginous fruits; miscellaneous grains, seeds and fruit; industrial or medicinal plants; straw and fodder	●
13	Lac; gums, resins and other vegetable sap and extracts	●
14	Vegetable plaiting materials and other vegetable products	●
15	Animal, vegetable fats and oils, cleavage products, etc.	●
16	Edible preparations of meat, fish, crustaceans, mollusks or other aquatic invertebrates	●
17	Sugars and sugar confectionary	●
18	Cocoa and cocoa preparations	●
19	Preparations of cereals, flour, starch or milk; bakers wares	●
20	Preparations of vegetables, fruit, nuts or other plant parts	●
21	Miscellaneous edible preparations	●
22	Beverages, spirits and vinegar	●
23	Food industry residues and waste; prepared animal feed	●
24	Tobacco and manufactured tobacco substitutes	●
25	Salt; sulfur; earth and stone; lime and cement plaster	●
26	Ores, slag and ash	●
27	Mineral fuels, mineral oils and products of their distillation; bitumin substances; mineral wax	●
28	Inorganic chemicals; organic or inorganic compounds of precious metals, of rare-earth metals, of radioactive elements or of isotopes	●
29	Organic chemicals	●
30	Pharmaceutcal products	●
31	Fertilizers	●
32	Tanning or dyeing extracts; tannins and derivatives; dyes, pigments and coloring matter; paint and varnish; putty and other mastics; inks	●
33	Essential oils and resinoids; perfumery, cosmetic or toilet preparations	●
34	Soap; waxes; polish; candles; modeling pastes; dental preparations with basic of plaster	●
35	Albuminoidal substances; modified starch; glues; enzymes	●
36	Explosives; pyrotechnic products; matches; pyrophoric alloys; certain combustible preparations	●
37	Photographic or cinematographic goods	●
38	Miscellaneous chemical products	●
39	Plastics and articles thereof	●
40	Rubber and articles thereof	●
41	Raw hides and skins (other than furskins) and leather	●
42	Leather articles; saddlery and harness; travel goods, handbags and similar; articles of animal gut (not silkworm gut)	●

43	Furskins and artificial fur; manufactures thereof	●
44	Wood and articles of wood; wood charcoal	●
45	Cork and articles of cork	●
46	Manufactures of straw, esparto or other plaiting materials; basketware and wickerwork	●
47	Pulp of wood or of other fibrous cellulosic material; waste and scrap of paper and paperboard	●
48	Paper and paperboard and articles thereof; paper pulp articles	●
49	Printed books, newspapers, pictures and other products of printing industry; manuscripts, typescripts	●
50	Silk, including yarns and woven fabric thereof	●
51	Wool and animal hair, including yarn and woven fabric	●
52	Cotton, including yarn and woven fabric thereof	●
53	Other vegetable textile fibers; paper yarn and woven fabrics of paper yarn	●
54	Manmade filaments, including yarns and woven fabrics	●
55	Manmade staple fibers, including yarns and woven fabrics	●
56	Wadding, felt and nonwovens; special yarns; twine, cordage, ropes and cables and article thereof	●
57	Carpets and other textile floor coverings	●
58	Special woven fabrics; tufted textile fabrics; lace; tapestries; trimmings; embroidery	●
59	Impregnated, coated, covered or laminated textile fabrics; textile articles for industrial use	●

60	Knitted or crocheted fabrics	●
61	Apparel articles and accessories, knitted or crocheted	●
62	Apparel articles and accessories, not knitted or crocheted	●
63	Other textile articles; needlecraft sets; worn clothing and worn textile articles; rags	●
64	Footwear, gaiters and the like and parts thereof	●
65	Headgear and parts thereof	●
66	Umbrellas, walking sticks, seat sticks, riding crops, whips, and parts thereof	●
67	Prepared feathers, down and articles thereof; artificial flowers; articles of human hair	●
68	Articles of stone, plaster, cement, asbestos, mica or similar materials	●
69	Ceramic products	●
70	Glass and glassware	●
71	Pearls, precious stones, metals, coins, etc.	●
72	Iron and steel	●
73	Articles of iron and steel	●
74	Copper and articles thereof	●
75	Nickel and articles thereof	●
76	Aluminum and articles thereof	●
77	Lead and articles thereof	●
78	Zinc and articles thereof	●
79	Tin and articles thereof	●
80	Other base metals; cermets; articles thereof	●
81	Tools, implements, cutlery, spoons and forks of base metal and parts thereof	●
82	Miscellaneous articles of base metal	●
83	Nuclear reactors, boilers, machinery and mechanical appliances; parts thereof	●

84	Electric machinery, equipment and parts; sound equipment; television equipment	●
85	Railway or tramway; locomotives, rolling stock, track fixtures and parts thereof; mechanical and electromechanical traffic signal equipment	●
86	Vehicles (not railway, tramway, rolling stock); parts and accessories	●
87	Aircraft, spacecraft, and parts thereof	●
88	Ships, boats and floating structures	●
89	Optical, photographic, cinematographic, measuring, checking, precision, medical or surgical instruments/apparatus; parts and accessories	●
90	Clocks and watches and parts thereof	●
91	Musical instruments; parts and accessories thereof	●
92	Arms and ammunition, parts and accessories thereof	●
93	Furniture; bedding, mattresses, cushions, etc.; other lamps and light fitting, illuminated signs and nameplates, prefabricate buildings	●
94	Toys, games and sports equipment; parts and accessories	●
95	Miscellaneous manufactured articles	●
96	Works of art, collectors pieces and antiques	●

Table 1: List of commodities, according to the standard international classification HS1996 [16], and associated codes, as provided by the BACI-Comtrade dataset [15]. In the third column we divide such commodities in classes of similar traded items, each of them being represented by a different colored circle. Colors are the same as reported in the dendrogram in Figure 1.

Code	Airline
1	NextJet
2	International Business Air
3	Widerøe
4	Orenburg Airlines
5	Balkan Bulgarian Airlines
6	Flybaboo
7	Carpatair
8	Twin Jet
9	Air Bosna
10	Airlinair
11	Corse-Mediterranee
12	Air Sicilia
13	Estonian Air
14	Israir
15	Travel Service
16	Atlantis European Airways
17	Vladivostok Air
18	Arkia Israel Airlines
19	Cargoitalia
20	Icelandair
21	Eastern Airways
22	Vueling Airlines
23	Hahn Air
24	Aeroflot-Nord
25	Aircompany Yakutia
26	Aerocondor
27	NordStar Airlines
28	Tatarstan Airlines
29	IzAvia
30	Moskovia Airlines
31	Gazpromavia
32	Aigle Azur
33	SATA International
34	SATA Air Acores
35	Monarch Airlines
36	Jet2.com
37	Scandinavian Airlines System
38	Azerbaijan Airlines
39	Onur Air
40	Air Armenia
41	Transavia Holland
42	Adria Airways
43	Teamline Air
44	Binter Canarias
45	Air Europa
46	Montenegro Airlines
47	Austrian Airlines
48	Luxair
49	Starling Airlines Spain
50	Olympic Airlines
51	Germanwings
52	Pegasus Airlines
53	Atlasjet
54	Belair Airlines
55	Air Moldova
56	Blue Panorama Airlines
57	Air One
58	Ryanair
59	Transavia France
60	Flybe
61	Wizz Air
62	Belavia Belarusian Airlines
63	Brussels Airlines
64	KLM Royal Dutch Airlines
65	Ural Airlines
66	North Flying
67	TAP Portugal
68	Alitalia
69	Aeroflot Russian Airlines
70	Air France
71	Norwegian Air Shuttle
72	British Airways
73	El Al Israel Airlines
74	Czech Airlines
75	Air Berlin
76	S7 Airlines
77	easyJet
78	Swiss International Air Lines
79	Rossiya-Russian Airlines
80	Transaero Airlines
81	Turkish Airlines
82	Condor Flugdienst
83	Aegean Airlines
84	Iberia Airlines
85	Ukraine International Airlines
86	LOT Polish Airlines
87	Cyprus Airways

88	Tarom
89	Germania
90	Bulgaria Air
91	Jat Airways
92	Niki
93	TUIfly
94	Air Malta
95	SunExpress
96	Meridiana
97	Aer Lingus
98	Air Baltic
99	Finnair
100	Croatia Airlines
101	Lufthansa
102	Air Dolomiti
103	DAT Danish Air Transport
104	Air Greenland
105	Atlantic Airways
106	AirOnix
107	Golden Air
108	Livingston
109	Maastricht Airlines
110	Air Iceland
111	Wizz Air Ukraine
112	BAL Bashkirian Airlines
113	Star1 Airlines
114	Eurolot
115	Malmö Aviation
116	Hex'Air
117	BAL Bashkirian Airlines
118	Intersky
119	Motor Sich
120	SmartLynx Airlines
121	Polet Airlines
122	Saratov Aviation Division
123	LTU International
124	Sat Airlines
125	Georgian Airways
126	UTair-Express
127	Air Europe
128	SkyWork Airlines
129	Danube Wings
130	JobAir

Table 2: List of European-based airlines operating in Europe, as provided by the OpenFlight database [17].

Acknowledgments

This work was supported by the EU project MULTIPLEX (contract 317532). DG also acknowledges support from the Netherlands Organization for Scientific Research (NWO/OCW) and the Dutch Econophysics Foundation (Stichting Econophysics, Leiden, the Netherlands) with funds from beneficiaries of Duyfken Trading Knowledge BV, Amsterdam, the Netherlands.

Author Contributions

V.G. analyzed the data and prepared the figures. D.G. planned the research. Both the authors wrote and reviewed the manuscript.

Additional information

Competing financial interests: The authors declare no competing financial interests.

References

[1] Bianconi, G. Statistical mechanics of multiplex networks: entropy and overlap. *Phys. Rev. E* **87**, 062806 (2013).

[2] Halu, A., Mukherjee, S. & Bianconi, G. Emergence of overlap in ensembles of spatial multiplexes and statistical mechanics of spatial interacting network ensembles. *Phys. Rev. E* **89**, 012806 (2014).

[3] Menichetti, G., Remondini, D., Panzarasa, P., Mondragón, R. J. & Bianconi, G. Weighted Multiplex Networks. *PLoS ONE* **9(6)**, e97857 (2014).

[4] Park, J. & Newman, M. E. J. Statistical mechanics of networks. *Phys. Rev. E* **70**, 066117 (2004).

[5] Garlaschelli, D. & Loffredo, M. I. Maximum likelihood: Extracting unbiased information from complex networks. *Phys. Rev. E* **78**, 015101 (2008).

[6] Squartini, T. & Garlaschelli, D. Analytical maximum-likelihood method to detect patterns in real networks. *New J. Phys.* **13**, 083001 (2011).

[7] Squartini, T., Mastrandrea, R., & Garlaschelli, D. Unbiased sampling of network ensembles. *arXiv:1406.1197* (2014).

[8] Garlaschelli, D. The weighted random graph model. *New J. Phys.* **11**, 073005 (2009).

[9] Maslov, S. & Sneppen, K. Specificity and stability in topology of protein networks. *Science* **296**, 910 (2002).

[10] Serrano, M. A. & Boguñá, M. Weighted configuration model. *AIP Conf. Proc.* **776**, 101 (2005).

[11] Squartini, T., Picciolo, F., Ruzzenenti, F. & Garlaschelli, D. Reciprocity of weighted networks. *Sci. Rep.* **3**, 2729 (2013).

[12] Garlaschelli, D. & Loffredo, M. I. Patterns of link reciprocity in directed networks. *Phys. Rev. Lett.* **93**, 268701 (2004).

[13] Barigozzi, M., Fagiolo, G. & Garlaschelli, D. Multinetwork of international trade: a commodity-specific analysis. *Phys. Rev. E* **81**, 046104 (2010).

[14] Mantegna, R. Hierarchical structure in financial markets. *Eur. Phys. J. B* **11**, 193 (1999).

[15] Gaulier, G. & Zignago, S. BACI: international trade database at the product-level. *CEPII Working Paper* **23** (2010).

[16] http://www.wcoomd.org

[17] http://openflight.org
